# A Treatment with a Protease Inhibitor Recombinant from the Cattle Tick (*Rhipicephalus Boophilus microplus*) Ameliorates Emphysema in Mice

**DOI:** 10.1371/journal.pone.0098216

**Published:** 2014-06-02

**Authors:** Juliana D. Lourenço, Luana P. Neves, Clarice R. Olivo, Adriana Duran, Francine M. Almeida, Petra M. M. Arantes, Carla M. Prado, Edna Aparecida Leick, Aparecida S. Tanaka, Mílton A. Martins, Sergio D. Sasaki, Fernanda D. T. Q. S. Lopes

**Affiliations:** 1 Department of Medicine, University of Sao Paulo, Sao Paulo, Brazil; 2 Biological Science Department, UNIFESP, Sao Paulo, Brazil; 3 Departamento de Bioquímica, UNIFESP-EPM, Sao Paulo, Brazil; 4 Centro de Ciências Naturais e Humanas, UFABC, Santo André, Sao Paulo, Brazil; Faculty of Animal Sciences and Food Engineering, University of São Paulo, Pirassununga, SP, Brazil, Brazil

## Abstract

**Aims:**

To determine whether a serine protease inhibitor treatment can prevent or minimize emphysema in mice.

**Methods:**

C57BL/6 mice were subjected to porcine pancreatic elastase (PPE) nasal instillation to induce emphysema and were treated with a serine protease inhibitor (rBmTI-A) before (Protocol 1) and after (Protocol 2) emphysema development. In both protocols, we evaluated lung function to evaluate the airway resistance (Raw), tissue damping (Gtis) and tissue elastance (Htis). The inflammatory profile was analyzed in the bronchoalveolar lavage (BALF) and through the use of morphometry; we measured the mean linear intercept (Lm) (to verify alveolar enlargement), the volume proportion of collagen and elastic fibers, and the numbers of macrophages and metalloprotease 12 (MMP-12) positive cells in the parenchyma. We showed that at both time points, even after the emphysema was established, the rBmTI-A treatment was sufficient to reverse the loss of elastic recoil measured by Htis, the alveolar enlargement and the increase in the total number of cells in the BALF, with a primary decrease in the number of macrophages. Although, the treatment did not control the increase in macrophages in the lung parenchyma, it was sufficient to decrease the number of positive cells for MMP-12 and reduce the volume of collagen fibers, which was increased in PPE groups. These findings attest to the importance of MMP-12 in PPE-induced emphysema and suggest that this metalloprotease could be an effective therapeutic target.

## Introduction

Chronic Obstructive Pulmonary Disease (COPD) is characterized by a chronic airflow limitation caused by small airway disease (obstructive bronchiolitis) and parenchymal destruction (emphysema). It is the fourth leading cause of death throughout the world, and further increases in the disease prevalence are predicted in the coming decades [1].

In emphysema there is an abnormal enlargement of the distal alveoli accompanied by the destruction of their walls without obvious fibrosis [1], and an imbalance between the proteases and anti-proteases still remains the principal hypothesis to explain the pathogenesis of this disease [2].

Although there are many clinical and experimental studies on the physiopathological mechanisms in emphysema, there are no effective pharmacotherapeutic strategies to inhibit the progression of alveolar wall destruction.

Emphysema in cigarette smokers is believed to be mediated by elastolytic proteases released by inflammatory cells that locally overwhelm or evade inhibitors, causing destruction of the elastin and other extracellular matrix proteins in the alveolar walls [3], [4].

There is no consensus on which specific cell(s) and which proteases are the most important to emphysema development. Both serine proteases and matrix metalloproteases are expressed in human emphysema, and animal models involving the instillation of neutrophil elastase [5] and knockout mice for metalloprotease 12 (MMP-12) [4] have been used to clarify the pathogenesis of this disease.

Some studies have shown that protease inhibitors have positive effects against emphysema progression in animal models [6], [7], [8]. Wright et al. [9] showed that guinea pigs exposed to cigarette-smoke either acutely or chronically and treated with a serine elastase inhibitor (ZD0892) showed a reduction in inflammatory activity and in parenchymal destruction. Kuraki et al. [10] demonstrated that prior treatment with an oral neutrophil elastase inhibitor (ONO-6818) could inhibit lung hemorrhage and the accumulation of neutrophils in the lung of rats at the acute phase of lung injury induced by human neutrophil elastase; in long term studies, the administration of the same inhibitor at 8 weeks after HNE (Human Neutrophil Elastase) prevented HNE-induced emphysema.

In the current study, we verified the effects of treatment with the Kunitz-type serine protease inhibitor from the cattle tick *Rhipicephalus (B.) microplus* (**rBmTI-A**) in an elastase-induced model in mice both before and after emphysema development.

The Kunitz type serine protease inhibitor is a canonical inhibitor of serine proteases which domain has molecular mass around 7000 Da with three disulfide bridges, characterizing a protein with a stabilized structure [11]. *Rhipicephalus (B.) microplus* is a very important bovine ectoparasite with an extensive geographic distribution in tropical and subtropical regions of the world, especially in Brazil [12]. In both the larvae and eggs of these ticks, a serine proteinase-inhibiting activity has been described that protects the host from infection by pathogens or parasites, inhibits fungal or bacterial proteinases, and which likely regulates proteinases involved in coagulation or cytokine activation [13].

Therefore, we postulated that treatment with a recombinant serine protease inhibitor (rBmTI-A) in the animals that received porcine pancreatic elastase (PPE) by nasal instillation could prevent or minimize emphysema development.

## Methods

This study was approved by the Review Board for human and animal studies of the School of Medicine of University of São Paulo (São Paulo, Brazil). Six- to eight-week old male C57BL/6 mice were used in this study. All the animals in the study received humane care in compliance with the Guide for the Care and Use of Laboratory Animals (NIH publication 85-23, revised 1985).

### Induction of emphysema

To induce emphysema, the animals received a nasal instillation of 50 µL (0.667 IU) of porcine pancreatic elastase (PPE) (6.6 units/mg, E-1250, Type I) (Sigma, St. Louis, MO) [14]. The control groups received 50 µL of 0.9% NaCl (saline solution), the PPE vehicle.

### Preparation of the rBmTI-A

#### BmTI-A cDNA determination

Engorged female *Rhipicephalus Boophilus microplus* ticks were provided by Dr. Itabajara da Silva Vaz Junior of Universidade Federal do Rio Grande do Sul, Brazil. *Pichia pastoris* (GS115) and the vector pPIC9K were purchased from Invitrogen (San Diego, CA).

The cDNA sequence encoding the native BmTI-A inhibitor, GenBank accession number P83609, isolated from a larval extract [15], was identified in the DFCI Gene Indices Information Page (accession number: TC20102). The sequence analyses were performed using the BLAST algorithm [16]. The alignment of the protein sequences was performed with the ClustalW program, version 1.83 [17].

#### Construction of pPIC9K-BmTI-A

The BmTI-A gene was amplified by PCR using B. microplus ovary cDNA as a template. The SnaBI and NotI restriction sites were added to the sense primer fBmTI-A (5′ GAAGCTTACGTATCGCAACCACATGTG AACCC3′) and anti-sense primer rBmTI-A (5′ AAAGAATGCGGCCGCTTATGA TTTCTTGCAGCTGTTTAGGC 3′), respectively. The gene was digested with the SnaBI and NotI restriction enzymes, and the fragment was ligated into the plasmid pPIC9K (Invitrogen, San Diego, CA). The resulting plasmid (pPIC9K-BmTI-A) was linearized with the SacI restriction enzyme that was used in the transformation of the competent P. pastoris GS115 yeast strain cells, prepared according to the manufacturer's instructions. rBmTI-A followed the methodology of Sasaki and Tanaka, 2008 [18]. The transformed yeast were incubated for 5 days in buffered methanol-complex medium (BMMY). After cultivation, the yeast cells were harvested by centrifugation (4000×g, 20 min, 4°C) and the supernatant containing the inhibitory activity was stored at −20°C.

#### The recombinant BmTI-A (rBmTI-A) purification

The purification of rBmTI-A expressed in the *P. pastoris* system was carried out using two chromatographic steps: affinity chromatography in a trypsin–Sepharose column and reverse-phase chromatography in a Sephasil Peptide C_8_ column (Amersham Biosciences, Uppsala, Sweden). The culture supernatant containing the rBmTI-A was applied to a trypsin–Sepharose column previously equilibrated with 50 mM Tris–HCl buffer, pH 8.0 (buffer A). The weakly bound proteins were washed out with buffer A containing 0.2 M NaCl, and the rBmTI-A was eluted with a 0.2 M KCl solution at pH 2.0. The eluted fractions were immediately neutralized using 1 M Tris–HCl buffer, pH 8.0. The fractions containing the rBmTI-A were pooled, dialyzed, lyophilized, suspended in buffer A and applied to a Sephasil Peptide C_8_ column.

To verify that the protease inhibitor treatment prevented or minimized the elastase-induced emphysema in mice, the treatment with rBmTI-A was performed using two different protocols with different time points, namely, before the emphysema development and 21 days after the PPE instillation, and in lungs with established emphysema.

### Experimental groups

In order to assess the therapeutic effects of rBmTI-A in mice emphysema model, animals were divided into groups according to the treatment protocols as follows:

*Protocol 1*: C57BL/6 mice (20–25 g) received either a nasal instillation of 50 µl (0.667 UI) of porcine pancreatic elastase (PPE) or normal saline (S), and 1 hour later the animals received a single dose of the protease inhibitor (rBmTI-A, 35.54 pmol, by nasal instillation) or vehicle (VE). The animals were divided in 4 experimental groups: S-VE (n = 7), S-rBmTIA (n = 10), PPE-VE (n = 8) and PPE-rBMTIA (n = 10).

*Protocol 2*: C57BL/6 mice (20–25 g) received either a nasal instillation of 50 µl (0.667 UI) of porcine pancreatic elastase (PPE) or normal saline (S), and 21 days later the animals received two doses of either a protease inhibitor (rBmTI-A, 35.54 pmol, by nasal instillation) or vehicle (VE) with a 7-day interval. The animals were divided in 4 experimental groups: S-VE (2 doses) (n = 10), S-rBmTIA (2 doses) (n = 8), PPE-VE (2 doses) (n = 9) and PPE-rBMTIA (2 doses) (n = 10).

### Respiratory mechanics

The animals were deeply anesthetized by an intraperitoneal injection of thiopental (70 mg/kg), tracheostomized and then connected to a ventilator for small animals (FlexiVent, SCIREQ, Scientific Respiratory Equipment, Montreal, Quebec). Also, they were paralyzed with pancuronium bromide (1 mg/Kg), and the anesthetic level was checked during the entire procedure.

Mice were mechanically ventilated with a tidal volume of 10 mL/kg and breathing frequency of 120 breaths/min. The respiratory system input impedance (Zrs) was measured by applying 3 s of oscillatory volume perturbation (frequencies from 0.25 to 9.125 Hz) to the tracheal cannula. To calculate the respiratory mechanics parameters of airway resistance (*Raw*), tissue damping (*G*) and tissue elastance (*H*) from Zrs data, we used the constant phase model described by Hantos et al. [19]:
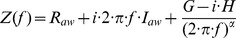



Where i is the imaginary component, f is frequency and 
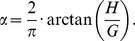



The Raw parameter reflects the airway components, while Gtis and Htis embody energy dissipation and storage, respectively, within the tissues [20].

### Bronchoalveolar Lavage Fluid (BALF)

At the end of the respiratory mechanics assessment, the animals were exsanguinated via the abdominal aorta and the BALF was collected. The trachea was cannulated and the BALF was obtained by washing the airway lumen with 3×0.5 mL of sterile saline. The recovery volume was over 95% of the instilled fluid and was put into a test tube on ice. The white blood cells was quantified by total and differential counting. The BALF was centrifuged at 800× for 10 min and the cell pellet was resuspended in 0.2 mL of sterile saline. The total number of viable cells was determined in a Neubauer hemocytometer counting chamber. The differential cell counts were performed in cytocentrifuge preparations of the BALF (450 rpm for 6 min) (Cytospin, Cheshire, UK) stained with Diff-Quick (Biochemical Sciences Inc., Swedesboro, NJ). At least 300 cells were counted according to standard morphologic criteria.

### Lung Biopsies Preparation

At the end of the respiratory mechanics evaluation, the abdominal wall was opened, and the animals were exsanguinated via the abdominal aorta. The thoracic cavity was then opened, and the lungs were removed. Both the lungs were fixed using 10% buffered formalin infused through the trachea at a constant pressure of 20 cm H_2_0 for 24 hours and then embedded in paraffin. Lung tissue sections (5 µm) were stained with H&E for lung structure analysis, using Sirius Red (for collagen fibers) and Resorcin-Fuchsin (for elastic fibers).

### Immunohistochemistry

The tissue sections were deparaffinized and hydrated. After blocking the endogenous peroxidase activity, an antigen retrieval step was performed with either high-temperature citrate buffer (pH = 6.0) or trypsin. The following primary antibodies were used in this study: a goat polyclonal anti-mouse MMP-12 (1:60, Santa Cruz Biotechnology, CA, USA) and an anti-mouse macrophage marker MAC-2 (1:30,000, clone M3/38, Cedarlane, ON, Canada). The Vectastain ABC Kit (Vector Laboratories, Burlingame, CA, USA) was used in conjunction with a species-specific secondary antibody, and 3-diaminobenzidine (DAB, Sigma, St. Louis, MO, USA) was used as the chromogen. The sections were counterstained with Harris's hematoxylin. As a negative control, the primary antibody was omitted from the procedure, and BSA was used instead.

### Morphometry

For conventional morphometry, an eyepiece with a coherent system of 50 lines, 100 points and a known area, which was attached to the microscope reticle, was used. The mean linear intercept (Lm), an indicator of the mean alveolar diameter [21], was assessed in 20 non-overlapping fields of the lung parenchyma per animal at 200× magnification. The volume proportion of collagen and elastic fibers in the alveolar parenchyma were determined using the same eyepiece. We counted the number of points hitting a specific fiber in the alveolar parenchyma and compared that with the number of points hitting the alveolar tissue in each field to generate each proportion at 400× magnification. The number of macrophages and the number of MMP-12-expressing cells in the alveolar parenchyma were also assessed by a point-counting technique. Using the eyepiece (62,500 µm^2^ at 400× magnification), the number of points in each field contacting the alveolar tissue was counted. The alveolar tissue area in each field was calculated as the number of points intersecting the alveolar tissue as a proportion of the total grid area.

### Statistical analysis

All of the data are expressed as the means and SD. A statistical analysis was performed using SigmaStat software (SPSS Inc. Chicago, Illinois, USA). The values were compared using a two-way ANOVA followed by all pairwise multiple comparison procedures (Holm-Sidak method). A *p*-value of less than 0.05 was considered to be significant.

## Results

### Respiratory mechanics

**Figure 1:**
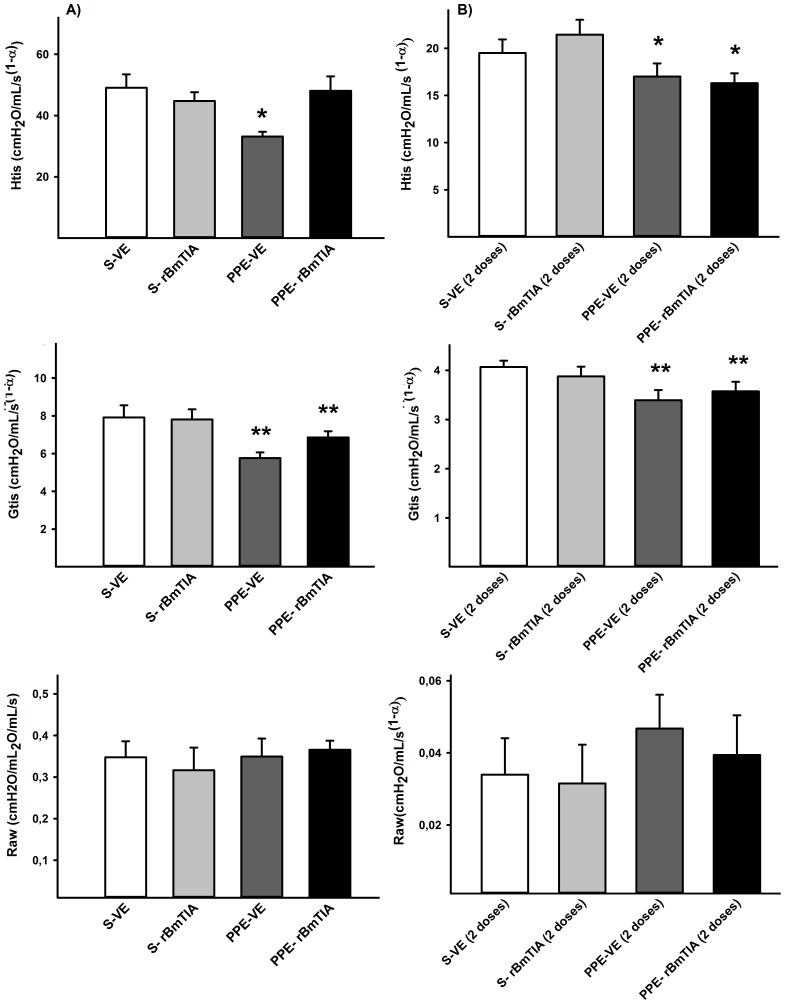
Htis, Gtis and Raw parameters in the first and second protocols are represented in Figures 1 A and B, respectively. A) * p = 0.032 compared to the other groups; ** p = 0.004 compared to S-VE and S- rBmTIA groups. B) * p = 0.009 compared to S-VE and S-rBmTIA groups; ** p = 0.011 compared to S-VE and S-rBmTIA groups. There were no significant differences in Raw values among the experimental groups. Values are means and SD.

Figure 1 shows the mean values and SD of the respiratory mechanics parameters. In the first protocol, there was a significant decrease in the Htis only in the PPE-VE group (p = 0.032), while the Gtis analysis showed significant differences between the PPE and S groups that were independent of the rBmTI-A treatment (p = 0.004). In the second protocol, there was a significant decrease in the Htis (p = 0.009) and Gtis (p = 0.011) values in both groups that received the PPE instillation compared with the S groups. We did not observe differences in the Raw values between the experimental groups.

### Bronchoalveolar Lavage Fluid (BALF)

**Figure 2:**
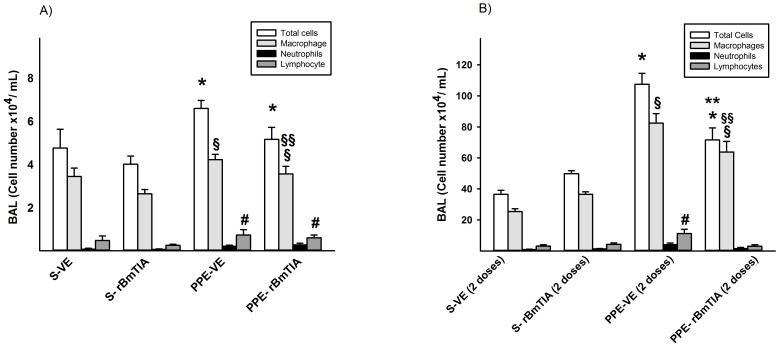
BALF analyses in the first and second protocols are shown in figures 2A and B, respectively. A) Total Cells: * p = 0.02 compared to S groups; Macrophages: ^§^ p = 0.02 compared to S groups and ^§§^ p = 0.004 compared to PPE-VE; Lymphocytes: ^#^ p = 0.03 compared to S groups. B) Total Cells: * p≤0.01 compared to S groups and ** p≤0.01 compared to PPE-VE group; Macrophages: ^§^ p = 0.05 compared to S Groups and ^§§^ p = 0.013 compared to PPE-VE group; Lymphocytes: ^#^p≤0.01 compared to the other groups. Values are means and SD.

In the first protocol, the bronchoalveolar lavage fluid analysis showed an increase in the total number of cells in both groups that received the PPE (p = 0.02). The numbers of macrophages (*p = 0.02) and lymphocytes (*p = 0.03) were increased in the PPE groups, and the treatment with rBmTI-A decreased only the number of macrophages (**p = 0.04) (Figure 2A). Additionally, in the second protocol, there was an increase in the total number of cells in the PPE groups (*p≤0.01), however there was a decrease in the PPE-rBmTIA compared with the PPE-VE group (**p p≤0.01). The differential count of the cells showed an increase in macrophages (*p = 0.005) and lymphocytes (*p≤0.01) in the PPE groups, and rBmTI-A treatment decreased the number of macrophages (**p = 0.013).

### Mean Linear Intercept (Lm)

**Figure 3:**
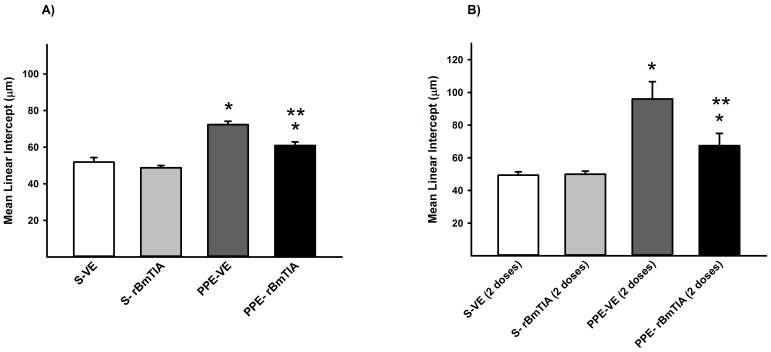
Mean linear intercept (Lm) values measured in the first (A) and second (B) protocols (means and SD). **A**) * p<0.001 compared to S-VE and S-rBmTIA groups; ** p<0.001 compared to PPE-VE group. B) * p<0.001 compared to S-VE and S- rBmTIA groups; ** p = 0.04 compared to PPE-VE group.

In the first (Figure 3A) and second protocols (Figure 3B), there was an increase in the Lm mean values and SD in the PPE groups compared with the S groups (*p = 0.037, 3A; and *p≤0.03, 3B), however the PPE-rBmTIA groups showed lower values compared with the PPE-VE groups (3A and 3B, **p≤0.001).

### Volume proportion of collagen and elastic fibers

**Figure 4:**
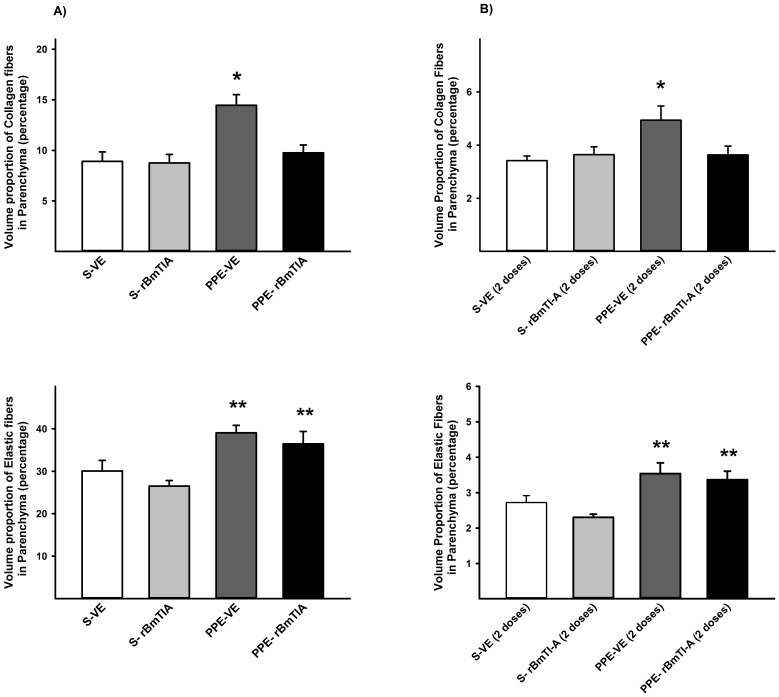
Volume proportion of collagen and elastic fibers are shown in the first (A) and second protocols (B). A) * p = 0.031 compared to the other groups; ** p≤0.001 compared to S groups; B) * p = 0.035 compared to the other groups; ** p = 0.0003 compared to S groups. Values are means and SD.

In Figure 4, the mean values and SD of the volume proportions of collagen fibers (A) and elastic fibers (B) are shown. There was an increase in collagen fibers in the PPE-VE groups compared with the others (*p<0.05), while the elastic fibers were increased in the PPE-VE and PPE-rBmTIA compared with the S groups (*p<0.05) in both protocols.

### Lung immunohistochemistry

**Figure 5:**
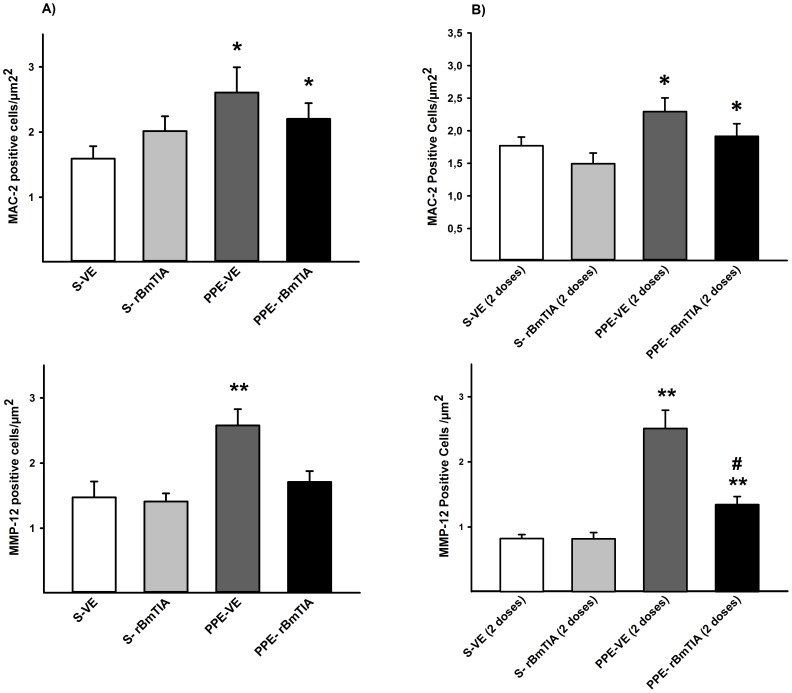
Positive cells for MAC-2 and MMP-12 in the first and second protocols (Figure 5A and B, respectively). A) * p = 0.037 compared to S-VE and S- rBmTIA groups; ** p = 0.024 compared to the other groups. B) * p = 0.011 compared to S-VE and S- rBmTIA groups; ** p = 0.003 compared to S-VE and S- rBmTIA groups; ^#^ p≤0.01 compared to PPE-VE group. Values are means and SD.

Although in the first protocol, the number of cells positive for MAC-2 was increased in the alveolar tissue of the mice that received the PPE nasal instillation (*p = 0.037), only the PPE-VE group showed higher values for MMP-12 (*p = 0.024) (Figure 5A). After the development of emphysema (Figure 5B), both groups that received the PPE instillation showed an increase in the number of MAC-2-positive (*p = 0.01) and MMP-12-positive (*p = 0.003) cells. However, the PPE-rBmTIA showed lower values compared with PPE-VE (*p<0.001).

## Discussion

In this study we evaluated the effects of a recombinant protease inhibitor treatment (rBmTI-A) before and after the development of emphysema in mice. The original protease inhibitor BmTI-A was not used, since its production requires a large amount of larvae ticks that are difficult to obtain in order to produce an enough amount of native inhibitor. Thus, we decided to use the strategy of cloning, expressing and purifying the recombinant inhibitor rBmTI-A. In addition, the recombinant inhibitor rBmTI-A presents the same inhibitory activities toward bovine trypsin, human neutrophil elastase (HNE), Human plasma Kallikrein (HuPK) and plasmin, all the Ki in nano molar rate. The recombinant inhibitor is a better inhibitor to HuPK and plasmin than the native inhibitor BmTI-A [15].

We found an effective decrease in alveolar enlargement at both time points, including 21 days after the PPE instillation, when the emphysema was already established. Since Lm is a measurement of the average space between opposing alveolar walls [21] and the emphysema is characterized by alveolar wall destruction [1], the Lm increase in PPE groups in both protocols suggests emphysema development in this experimental model and that the rBmTI-A treatment decreased these parenchymal lesions.

Additionally, the inflammatory profile analysis in the BALF showed a decrease in the total number of cells after the protease inhibitor treatment, with a decrease in the number of macrophages at the different time points.

In COPD patients, macrophages are predominant in the bronchoalveolar lavage and are believed to play an important role in the underlying inflammation in the distal airways [22], [23], [24]. It was demonstrated in resected human lungs that the extent of emphysema was directly related to an increase in the number of macrophages but not of neutrophils [25].

Although the morphometric analysis of the lung parenchyma showed that the treatment with rBmTI-A did not control the increase in macrophages either before or after the development of emphysema, we observed that the treatment with this inhibitor was sufficient to prevent an increase in the number of MMP-12 positive cells when it was administered 1 h after the emphysema induction by PPE nasal instillation. Even though the rBmTI-A treatment administered after emphysema development resulted in a decrease in MMP-12 positive cells in the PPE-rBMTIA compared with the PPE-VE groups, the PPE-rBMTIA group continued to have higher values compared with the control groups.

Since Hautamaki et al. [26] reported that knockout mice for MMP-12 did not develop emphysema after exposure to cigarette smoke, MMP-12 has been described as a metalloprotease released mainly by macrophages, and it is suggested as an important elastolytic enzyme responsible for emphysematous lesions in rodents. In our study, the rBmTI-A treatment was sufficient to reduce the number of cells positive for MMP-12, which could explain the inhibitory effects of rBmTI-A on the parenchymal destruction.

In emphysema, the progressive chronic inflammatory response in the lung tissues is associated with a dynamic tissue repair and remodeling process, which involves a structural reorganization of the extracellular matrix (ECM) components [27], such as collagen and elastic fibers. This could alter the lung viscoelastic properties, as evaluated by respiratory mechanics analysis, such as the tissue damping (Gtis) and tissue elastance (Htis) [28], [29], [30].

The respiratory mechanics analysis showed that the rBmTI-A treatment was sufficient to reverse the loss of elastic recoil measured by Htis in the PPE animals that received the protease inhibitor treatment at both time points, suggesting that the rBmTI-A instillation prevented and minimized the impairment of lung function in these animals.

Our analysis of the elastic and collagen fibers revealed an increase in the total numbers of both types of fibers in the PPE compared with the control groups, and the rBmTI-A instillation treatment was effective in reducing only the increase in collagen fibers in the PPE groups at both time points; rBmTI-A did not reduce the observed increase in the elastic fibers.

It is interesting that despite the increase in the number of elastic fibers, we observed an improvement in lung function in the animals that received the PPE instillation and rBmTI-A treatment. Many studies have shown that in parenchymal lung injury, as in our experimental model, the repair of elastic fibers is most likely defective, resulting in non-functional fibers.

Elastic fibers are considered to be the major components responsible for the elastic recoil properties of the lungs [31], [32], and they are composed of elastin and microfibrils (fibrillins, microfibril-associated glycoproteins, and TGF-β binding proteins) [31]. Fibrillin fibers assist in the formation of elastin polymers by providing a scaffold that directs elastin aggregation. The mice lacking elastin or elastic fiber proteins, such as fibrillin-1, show an emphysema-like lung at birth [33], [34], [35]. In a previous study we showed an increase in elastin in the PPE-exposed animals 28 days after the emphysema induction with no increase in type I fibrillin [36].

To better correlate our respiratory mechanics assessment results with the fiber amount analysis, further investigations will be necessary to evaluate which fiber types are present in greater numbers in the animals with emphysema after the treatment with the rBmTI-A and how these increased elastic fibers are arranged into fibrillin and elastin subcomponents.

The rBmTI-A has been described as an inhibitor of neutrophil elastase, trypsin and kallikrein. Until now, there has been no description in the literature of the inhibitory effects of rBmTI-A on MMP-12, or on any MMPs for that matter.

MMPs are generally released as latent precursors and the proteolytic cleavage of the latent forms results in active proteases [37]. Zhu et al. [37] demonstrated in a three-dimensional (3D) collagen gel culture that monocytes and fibroblasts can release MMP-1, -2, -3 and -9 and can degrade extracellular matrix in the presence of neutrophil elastase. It is possible that, in our study, these positive effects after the rBmTI-A treatment to minimize and prevent emphysema in a PPE-induced model could be due to an inhibition in neutrophil elastase and a consequent lack of MMP-12 activation.

These findings attest to the importance of MMP-12 in PPE-induced emphysema and suggest that this metalloprotease could be an effective target for therapy.

## References

[1] Global Strategy for Diagnosis, Management, and Prevention of COPD, Updated 2013. Available: http://www.goldcopd.org/uploads/users/files/GOLD_Report_2013_Feb20.pdf. Accessed: 2013 Nov 06.

[2] Barnes PJ (2000) Chronic Obstructive Pulmonary Disease. N Eng J Med (Suppl 4): 269–280 10.1056/NEJM200007273430407 10911010

[3] Wright J, Farmer S, Churg A (2002) Synthetic serine elastase inhibitor reduces cigarette smoke-induced emphysema in guinea pigs. Am J Respir Crit Care Med 166: 954–960. 12359653 10.1164/rccm.200202-098OC

[4] Shapiro SD (2002) Proteinases in chronic obstructive pulmonary disease. Biochem Soc Trans (Suppl 2): 98–102. 10.1042/12023833

[5] Gross P, Pfitzer EA, Toker A, Babyak MA, Kaschak M (1965) Experimental emphysema: its production with papain in normal and silicotic rats. Arch Environ Health 11: 50–58. 14312390 10.1080/00039896.1965.10664169

[6] Senior RO, Anderson NR (1998) Chronic obstructive pulmonary disease. A J Respir Crit Care Med 157: 139–147. 10.1164/ajrccm.157.4.nhlbi-12 9563773

[7] Takayama M, Ishibashi M, Ishii H, Kuraki T, Nishida T, et al (2001) Effect of neutrophil elastase inhibitor (ONO-5046) on lung injury after intestinal ischemia-reperfusion. J Appl Physiol 91: 1800–1807. 11568165 10.1152/jappl.2001.91.4.1800

[8] Stockley RA (1998) Protease/antiproteases: pathogenesis and role in therapy. Clin Pulm Med 5: 203–210.

[9] Wright J, Farmer S, Churg A (2002) Synthetic serine elastase inhibitor reduces cigarette smoke-induced emphysema in guinea pigs. Am J Respir Crit Care Med 166: 954–960. 12359653 10.1164/rccm.200202-098OC

[10] Kuraki T, Ishibashi M, Takayama M, Shiraishi M, Yoshida M (2002) A novel oral neutrophil elastase inhibitor (ONO-6818) inhibits human neutrophil elastase-induced emphysema in rats. Am J Respir Crit Care Med 166: 496–500. 12186827 10.1164/rccm.2103118

[11] Otlweski J, Jelen F, Zakrzewska M, Oleksy A (2005) The many faces of protease-protein inhibitor interaction. The EMBO Journal 24(7): 1303–1310. 15775973 10.1038/sj.emboj.7600611 PMC1142537

[12] Willadsen P, Jongejan F (1999) Immunology of the Tick-Host Interaction and the Control of Ticks and Tick-borne Diseases. Parasitol Today 15: 258–262. 10377526 10.1016/s0169-4758(99)01472-6

[13] Kanost MR (1999) Serine proteinase inhibitors in arthropod immunity. Dev Comp Immunol 23: 291–301. 10426423 10.1016/s0145-305x(99)00012-9

[14] Ito S, Ingenito EP, Brewer KK, Lauren DB, Harikrishnan P, et al (2005) Mechanics, nonlinearity, and failure strenght of lung tissue in a mouse model of emphysema: possible role of collagen remodeling. J Appl Physiol 98: 503–11. 15465889 10.1152/japplphysiol.00590.2004

[15] Tanaka AS, Andreotti R, Gomes A, Torquato RJ, Sampaio MU, et al (1999) A double headed serine proteinase inhibitor – human plasma kallikrein and elastase inhibitor – from Boophilus microplus larvae. Immunopharmacology 45: 171–177. 10615008 10.1016/s0162-3109(99)00074-0

[16] Altschul SF, Madden TL, Schaffer AA, Zhang J, Zhang Z, et al (1997) Gapped BLASTand PSI-BLAST: a new generation of protein database search programs. Nucleic Acids Res 25: 3389–3402. 9254694 10.1093/nar/25.17.3389 PMC146917

[17] Thompson JD, Higgins DG, Gibson TJ (1994) CLUSTAL W: improving the sensitivity of progressive multiple sequence alignment through sequence weighting, position-specific gap penalties and weight matrix choice. Nucleic Acids Res 22: 4673–4680. 7984417 10.1093/nar/22.22.4673 PMC308517

[18] Sasaki SD, Tanaka AS (2008) rBmTI-6, a Kunitz-BPTI domain protease inhibitor from the tick Boophilus microplus, its cloning, expression and biochemical characterization. Vet Parasitol 155(1-2): 133–141. 18502587 10.1016/j.vetpar.2008.03.031

[19] Hantos Z, Daróczy B, Suki B, Nagy S, Fredberg JJ (1992) Input impedance and peripheral inhomogeneity of dog lungs. J. Appl. Physiol 72(1): 168–178. 10.1152/jappl.1992.72.1.168 1537711

[20] Gomes RFM, Shen X, Ramchandani R, Tepper RS, Bates JHT (2000) Comparative respiratory system mechanics in rodents. J ApplPhysiol 89: 908–916. 10.1152/jappl.2000.89.3.908 10956333

[21] Margraf LR, Tomashefski JF, Bruce MC, Dahms BB (1991) Morphometric analysis of the lung in bronchopulmonary dysplasia. Am Rev Respir Dis 143: 391–400. 1990959 10.1164/ajrccm/143.2.391

[22] Keatings VM, Evans DJ, O'Connor BJ, Barnes PJ (1997) Cellular profiles in asthmatic airways: a comparison of induced sputum, bronchial washings, and bronchoalveolar lavage fluid. Thorax 52: 372–374. 9196522 10.1136/thx.52.4.372 PMC1758529

[23] Martin TR, Raghu G, Maunder RJ, Springmeyer SC (1985) The effects of chronic bronchitis and chronic air-flow obstruction on lung cell populations recovered by bronchoalveolar lavage. Am Rev Respir Dis 132: 254–260. 4026050 10.1164/arrd.1985.132.2.254

[24] Boyle JR, McDermott E, Crowther M, Wills AD, Bell PR, et al (1998) Doxycycline inhibits elastin degradation and reduces metalloproteinase activity in a model of aneurysmal disease. J Vasc Surg 27: 354–361. 9510291 10.1016/s0741-5214(98)70367-2

[25] Finkelstein R, Fraser RS, Ghezzo H, Cosio MG (1995) Alveolar inflammation and its relation to emphysema in smokers. Am JRespir Crit Care Med 152: 1666–1672. 7582312 10.1164/ajrccm.152.5.7582312

[26] Hautamaki RD, Kobayashi DK, Senior RM, Shapiro SD (1997) Requirement for macrophage elastase for cigarette smoke-induced emphysema in mice. Science 277: 2002–2004. 9302297 10.1126/science.277.5334.2002

[27] Abraham T, Hogg J (2000) Extracellular matrix remodeling of lung alveolar wall in three dimensional space identified using second harmonic generation and multiphoton excitation fluorescence. J Structural Biol (Suppl 2): 189–196. 10.1016/j.jsb.2010.04.006 20412859

[28] Bates JHT, Abe T, Romero PV, Sato J (1989) Measurement alveolar pressure in close chest dogs during flow interruption. J. Appl. Physiol 67: 488–492. 10.1152/jappl.1989.67.1.488 2759977

[29] Gomes RFM, Shen X, Ramchandani R, Tepper RS, Bates JHT (2000) Comparative respiratory system mechanics in rodents. J Appl Physiol 89: 908–916. 10956333 10.1152/jappl.2000.89.3.908

[30] Faria AC, Costa AA, Lopes AJ, Jansen JM, Melo PL (2010) Forced oscillation technique in the detection of smoking-induced respiratory alterations: diagnostic accuracy and comparison with spirometry. Clinics (Suppl 5): 443–450. 10.1590/S1807-59322010001200012 PMC3020340 21340218

[31] Robbesom AA, Koenders MMJF, Smits NC, Hafmans T, Versteeg EMM, et al (2008) Aberrant fibrilin-1 expression in early emphysematous human lung: a proposed predisposition for emphysema. Modern Pathology 21: 297–307. 18084245 10.1038/modpathol.3801004

[32] Shifren A, Mecham RP (2006) The stumbling block in lung repair of emphysema: elastic fiber assembly. Proc Am Thorac Soc 3: 428–433. 16799087 10.1513/pats.200601-009AW PMC2658707

[33] Neptune ER, Frischmeyer PA, Arking DE, Myers L, Bunton TE, et al (2003) Dysregulation of TGFbeta activation contributes to pathogenesis in Marfan syndrome. Nat Genet 33: 407–411. 12598898 10.1038/ng1116

[34] Siracusa LD, McGrath R, Ma Q, Moskow JJ, Manne J, et al (1996) A tandem duplication within the fibrillin-1 gene is associated with the mouse tight skin mutation. Genome Res 6: 300–313. 8723723 10.1101/gr.6.4.300

[35] Zacchigna L, Vecchione C, Notte A, Cordenonsi M, Dupont S, et al (2006) Emilin1 links TGF-beta maturation to blood pressure homeostasis. Cell 124: 929–942. 16530041 10.1016/j.cell.2005.12.035

[36] Lopes FD, Toledo AC, Olivo CR, Prado CM, Leick EA, et al.. (2013) A comparative study of extracellular matrix remodeling in two murine models of emphysema. Histol Histopathol (Suppl 2): 269–276. 10.14670/HH-28.269 23275309

[37] Zhu Y, Liu X, Sköld CM, Wang H, Kohyama T, et al (2001) Collaborative interactions between neutrophil elastase and metalloproteinases in extracellular matrix degradation in tree-dimensional collagen gels. Respir Res 2: 300–305. 11686900 10.1186/rr73 PMC59520

